# Gambling and Impulsivity Traits: A Recipe for Criminal Behavior?

**DOI:** 10.3389/fpsyt.2018.00006

**Published:** 2018-01-29

**Authors:** Gemma Mestre-Bach, Trevor Steward, Roser Granero, Fernando Fernández-Aranda, María Teresa Talón-Navarro, Àngel Cuquerella, Marta Baño, Laura Moragas, Amparo del Pino-Gutiérrez, Neus Aymamí, Mónica Gómez-Peña, Núria Mallorquí-Bagué, Cristina Vintró-Alcaraz, Pablo Magaña, José Manuel Menchón, Susana Jiménez-Murcia

**Affiliations:** ^1^Pathological Gambling Unit, Department of Psychiatry, Institut d’Investigació Biomédica de Bellvitge (IDIBELL), Bellvitge University Hospital, Barcelona, Spain; ^2^Ciber Fisiopatología Obesidad y Nutrición (CIBERObn), Instituto de Salud Carlos III, Madrid, Spain; ^3^Department de Psicobiologia i Metodologia, Autonomous University of Barcelona, Barcelona, Spain; ^4^Department of Clinical Sciences, Faculty of Medicine, University of Barcelona, Barcelona, Spain; ^5^Institute of Legal Medicine and Forensic Sciences of Catalonia, Barcelona, Spain; ^6^Nursing Department of Mental Health, Public Health, Maternal and Child Health, Nursing School, University of Barcelona, Barcelona, Spain; ^7^Department of Law, Pompeu Fabra University, Barcelona, Spain; ^8^Ciber de Salud Mental (CIBERSAM), Instituto de Salud Carlos III, Madrid, Spain

**Keywords:** gambling disorder, impulsivity, criminal behavior, psychopathology, risk factors

## Abstract

Gambling disorder (GD) is a psychiatric condition that was recently recategorized as a non-substance-related addiction in the Diagnostic and Statistical Manual of Mental Health Disorders. Criminal activity is commonly associated with gambling; however, few empirical studies to date have examined sociodemographic and psychological variables in this population. In this study, we explored criminal behavior history in a sample of consecutively recruited treatment-seeking gamblers (*n* = 382) and compared subjects with a history of illegal acts (*n* = 103, 26.9%) to those with no criminal record (*n* = 279, 73.1%). Impulsivity and personality traits were specifically explored, along with other gambling-related severity factors. We found that gamblers who engaged in illegal activity were more likely to endorse high levels of urgency (i.e., the tendency to act out when experiencing heightened emotional states) and increased lack of premeditation. Gamblers with a history of criminal behavior also had greater GD severity levels and gambling-related debts. Additionally, these gamblers reported lower levels of self-directedness, which is characterized by difficulty in establishing and redirecting behavior toward one’s goals. Likewise, gamblers who had conducted criminal acts showed a tendency to engage in greater risk-taking behavior. These results shed new light on this understudied population and provide insights for developing targeted harm-prevention interventions and treatment protocols.

## Introduction

### Gambling Disorder (GD) Conceptualization

Gambling disorder is characterized by a maladaptive pattern of gambling behavior that persists despite negative consequences in major areas of life functioning. It was recently recategorized as a non-substance-related addiction in the Diagnostic and Statistical Manual of Mental Health Disorders (DSM-5) (1). This disorder more frequently occurs in men (2) and is often characterized by specific personality traits, high impulsivity levels, and cognitive distortions, such as illusion of control (3–5).

One of the DSM-IV-TR diagnostic criteria for pathological gambling (6) included carrying out criminal acts in order to support gambling behavior. However, after much debate, the scientific community considered that this criterion provided little accuracy, leading to the removal of the “illegal acts” criterion from DSM-5 (1). Many researchers in the field of criminology believe that committing criminal offenses in order to finance gambling behavior should be considered as an indicator of disorder severity, instead of as an independent diagnostic criterion (7, 8). Moreover, it has been argued that GD-related criminal acts seldom occur in the absence of other GD criteria (9). However, the clinical and societal importance of this criterion has been subject to considerable discussion (10). After a classification and regression tree analysis, Themcheff et al. (11) highlighted that the “illegal acts” criterion showed high discriminative capacity between social and problem gamblers, and suggested that policy makers take this information into account. Nonetheless, this framework requires additional empirical support before informed decisions can be made.

### Criminal Behavior Related to GD

The self-reported prevalence of criminal behaviors in individuals diagnosed with GD ranges from 14 to 30% (8, 12). This relatively high mismatch between results could be explained bearing in mind that crime and GD are related in a complex and multifactorial way, including high comorbidity with other disorders, the presence of associated risk behaviors, sociodemographic factors, and gambling-related circumstances (e.g., financial debts) (12–14). In an attempt to coalesce a functional theoretical framework, most of the existing body of research on this topic has focused on two main associations between these factors (15). On one hand, gambling behaviors could be part of a criminal lifestyle, related to antisocial personality disorder (16); on the other, criminal activity could be precipitated by GD, especially when money becomes scarce (13). Data suggest that the latter is more habitual, since individuals with GD usually do not have a criminal record or a history of norms transgression prior to developing gambling problems (17).

When considered within the framework of the general strain theory, gamblers who face negative events or emotions, such as extreme financial difficulties, might be more prone to turn to illegal activity to support their habit (18, 19). Likewise, these difficulties could also subsequently increase the probability of carrying out illegal acts in order to try to relieve financial hardships (20). GD-related crimes are frequently reported as being committed in desperation in order to amend financial predicaments brought about by gambling-related losses, or, in some cases, to fund additional gambling episodes (21).

Nevertheless, not all individuals with GD and financial burdens engage in criminal behavior. Several attempts have been made to explain the risk factors associated with GD-related crime in greater depth. For example, substance abuse has been found to be prevalent in patients with GD (14, 22). This frequent comorbidity adds another complex factor as to why gamblers may commit crimes, although no longitudinal studies to date have established a causal relationship between substance abuse and gambling-related criminal acts. Results from another study suggested that stimulant substance abuse may potentially facilitate gambling-related illegal acts due to their disinhibitory effects (12). Similarly, GD severity positively correlates, in most cases, with the occurrence of criminal behaviors (23). Therefore, engagement in criminal acts to support one’s gambling behavior is, in all likelihood reflective of GD severity reaching its nadir (8, 12, 21, 24, 25). During early stages of the disorder, crime is commonly reported to be carried out with remorse, and gamblers often claim that they have the intention of returning fraudulently obtained goods when their debts, derived from gambling behavior, have been settled. This logic for justifying criminal behavior greatly differs from others who commit crimes such as petty theft or fraud (26). However, when GD is consolidated and debts are increased, an individual with GD has more difficulties regulating their behavior according to their basic moral principles and signs of repentance are blurred (21, 27).

In addition to GD comorbidity and other clinical factors, sociodemographic and personality features are also associated with crime (12). One study identified different subtypes of GD patients who committed crimes, taking sociodemographic variables, personality traits and clinical information related to GD into account (28). Psychopathology levels and poor impulse control were some of the main characteristics that best distinguished GD groups with a criminal record. Although some findings in the criminology literature have suggested that GD patients present different typologies of criminal behavior, obtaining money to finance gambling behavior is usually the primary motive for these crimes (29). Specifically, the most common criminal offenses in this population are petty theft, theft, fraud and forgery (30). GD patients do not usually show a propensity for violent behavior; however, financially motivated violent crimes do occasionally occur in this population (31).

Assuming the “generality of deviance” perspective (32), which suggests that varied forms of risk-taking behaviors tend to cooccur among individuals, the spectrum of deviant and criminal behaviors appears to have a common denominator: the tendency to seek immediate reward or relief without concern for long-term negative consequences (33). Therefore, the authors suggest that self-control is a main factor in determining the likelihood of engaging in criminal acts (34). These behavioral patterns, such as personality traits associated with risk (sensation seeking, impulsivity and low self-control) and multiple domains of risky attitudes, are also common in patients with GD (35–37). The authors highlight the existence of a key wedge factor of common variance “the generality of deviance” in gamblers, suggesting that shared personality traits, such as greater risk taking, may be a driver of deviant behavior (38). In this vein, Mishra et al. (39) suggested that GD was strongly associated with progambling and risk-taking attitudes.

Impulsivity is increasingly understood to be an early risk factor for the development of both GD (40) and delinquency (41). Impulsivity is a multidimensional construct encompassing facets such as the dysregulation of outward behavior due to decreased inhibitory control or a prejudicial decision-making style (e.g., choosing immediate gratification over larger, delayed rewards) (35). In recent years, the UPPS-P framework of impulsivity has become one of the most utilized models of impulsivity in psychiatric research. This questionnaire divides impulsivity levels into five subscales: lack of premeditation, lack of perseverance, positive and negative urgency, and sensation seeking (42). Specifically, urgency, defined as emotionally charged impulsive behaviors in response to positive or negative moods, has been found to be crucial in distinguishing between clinically dysfunctional GD patients and recreational gamblers (43).

During adolescence (the age at which most individuals begin to gamble) (44), cognitive impulsivity has also been found to be associated with a more rapid acceleration into criminal behavior (41). Likewise, urgency and lack of premeditation are known to significantly correlate with each other in adolescents (45). Researchers have also observed that an impulsive decision-making style and high levels of urgency are associated with an increased acceptance of erroneous beliefs (e.g., believing that a series of losses must be followed by a win) during gambling behavior, thereby worsening economic consequences (46, 47). Given that gamblers encompass a very heterogeneous group of patients, one might postulate that gambling-related illegal acts could be more commonplace in younger, impulsive gamblers than in older gamblers whose gambling motivations might be driven by altered emotion regulation capacity (29, 35). To our knowledge, however, no studies to date have examined the role that impulsivity plays in criminal behavior within the context of gambling.

### GD, Criminal Behavior, and the Spanish Court System

Within Spanish civil law/civil code, legal mechanisms exist which aim to limit the capacity of an individual with GD to inflict financial damage onto themselves or others. Namely, revoking legal guardianship or declaring civil incapacity allows for capital losses resulting from GD to be protected (48). Similarly, GD patients have the option to voluntarily bar themselves access to gambling establishments, either online or land-based, as part of a state-sponsored harm reduction program. Enrollment in the program can be indefinite; although participants may opt out of it at any time.

The Spanish Criminal Code does not specifically mention gambling as a mitigating or extenuating circumstance capable of reducing the gravity of an offense with regards to sentencing or moral opprobrium. However, in practice, the Spanish court system tends to apply discretion by imposing minimum penalties in cases characterized by reduced freewill that exhibit a clear causal relationship between the committed crime and gambling addiction (17).

### Aims and Hypothesis

The primary aim of this study was to compare impulsivity traits in a sample of treatment-seeking GD patients who committed illegal acts to those who did not. Furthermore, we aimed to explore differences between these groups in terms of sociodemographic and psychological variables, and the type of illegal act committed in order to ascertain which variable(s) best predicted the presence of a history of criminal behavior.

As stated above, high levels of debt and significant financial problems because of gambling behavior is often indicated a primary motive for committing a crime (21); therefore, we hypothesized that the GD patients with a history of criminal behavior would present higher levels of debt than those without a criminal record. We also hypothesized that GD patients with a history of criminal behavior would be characterized by greater levels of GD severity, impulsivity, and overall psychopathology (8, 12). Likewise, we hypothesized that those gamblers with a history of committing multiple offenses would present increased psychopathology, GD severity and levels of accumulated debt (49).

## Materials and Methods

### Participants and Procedure

The sample consisted of 382 patients with a diagnosis of GD who were being treated at the Gambling Disorder Unit within the Department of Psychiatry at Bellvitge University Hospital (Barcelona, Spain). This public hospital is certified as a tertiary care center for the treatment of addictive behaviors and oversees the treatment of very complex cases. Patients were derived to the Bellvitge University Hospital Gambling Disorder Unit through general practitioners or *via* another healthcare professional; some patients were derived from prison health services, though their treatment was not compulsory in the majority of cases. Nonetheless, in a few cases, a judge may have dictated the need for specific GD treatment at our unit. All treatment services for GD within the public Spanish healthcare system are provided free of charge.

Sociodemographic, clinical and criminal additional information was taken, and patients individually completed all the questionnaires required for this study (requiring approximately 2 h) before initiating outpatient treatment. Only patients who sought treatment for GD as their primary mental health concern and who met DSM-5 criteria for GD (1) were included in our sample. Exclusion criteria were: the presence of an organic mental disorder, intellectual disability, a neurodegenerative condition, such as Parkinson’s disease, or an active psychotic disorder. Participants were classified in two groups according the presence (*n* = 279) or absence (*n* = 103) of criminal behaviors related to GD. Criminal behavior was assessed *via* a structured interview with a staff clinical psychologist.

The present study was carried out in accordance with the latest version of the Declaration of Helsinki. The University Hospital of Bellvitge Ethics Committee of Clinical Research approved the study, and written informed consent was obtained from all participants.

### Measures

#### GD Severity

##### DSM-5 Criteria (1)

Patients were diagnosed with pathological gambling if they met DSM-IV-TR criteria (6). It should be noted that with the release of the DSM-5 (1), the term pathological gambling was replaced with GD. All patient diagnoses were reassessed and recodified *post hoc* and only patients who met DSM-5 criteria for GD were included in our analysis.

##### South Oaks Gambling Screen (SOGS) (50)

This self-report 20-item screening questionnaire discriminates between probable pathological, problem and non-problem gamblers. The Spanish validation used in this work showed excellent internal consistency (α = 0.94) and test–retest reliability (*r* = 0.98) (51).

#### Impulsivity Traits

##### Impulsive Behavior Scale (UPPS-P) (52)

The UPPS-P measures five facets of impulsive behavior through self-report on 59 items: negative urgency; positive urgency; lack of premeditation; lack of perseverance; and sensation seeking. Individuals are asked to consider acts/incidents during the last 6 months when rating their behavior and attitudes. The Spanish-language adaptation shows good reliability (Cronbach’s α between 0.79 and 0.93) and external validity (53).

#### Psychopathology

##### Symptom Checklist-Revised (SCL-90-R) (54)

This is a 90-item questionnaire measuring psychological distress and psychopathology. The items assess nine symptom dimensions: somatization, obsessive-compulsive, interpersonal sensitivity, depression, anxiety, hostility, phobic anxiety, paranoid ideation, and psychoticism. The global score [Global Severity Index (GSI)] is a widely used index of psychopathological distress and was the only variable from this questionnaire used in this study. The Spanish adapted version was used in this study (55).

#### Personality

##### Temperament and Character Inventory-Revised (TCI-R) (56)

The TCI-R is a reliable and valid 240-item questionnaire measured on a 5-point Likert-type scale to evaluate personality traits. It is structured using seven primary personality dimensions: four temperamental factors (novelty seeking, harm avoidance, reward dependence, and persistence) and three character dimensions (self-directedness, cooperativeness, and self transcendence). The Spanish revised version used in this study (57) showed adequate internal consistency (Cronbach’s alpha a mean value of 0.87).

#### Alcohol and Other Drugs use-abuse

##### Alcohol Use Disorders Identification Test (AUDIT) (58)

This test was developed as a simple screening method for excessive alcohol consumption. Internal consistency has been found to be high, and test–retest data have suggested a high reliability (0.86) and a sensitivity of around 0.90. Specificity in different settings and for different criteria averages 0.80 or more (59). In this work, cutoff points of 8 and 20 were used to identify individuals with alcohol abuse and alcohol dependence, respectively (60).

##### Drug Use Disorders Identification Test (DUDIT) (61)

The DUDIT is an 11-item screening instrument developed to identify non-alcohol drug use patterns and various drug-related problems in the general public, as well as in individuals in clinical settings who are likely to meet criteria for a substance dependence diagnosis (61). The first nine items are scored on a 5-point Likert scale ranging from 0 to 4, and the last two are scored on 3-point scales (values of 0, 2, 4). Total scores can range from 0 to 44, with higher scores being indicative of a more severe drug problem. The following risk levels have been suggested for DUDIT scores: no drug-related problems (total scores 0–5/1); possible drug-related problems, that is, risky or harmful drug habits that might be diagnosed as substance abuse/harmful use or dependence (6/2–24); likely heavily dependent on drugs (scores ≥ 25) (61).

#### Other Sociodemographic and Clinical Variables

Additional demographic, clinical, and social/family variables related to gambling were measured using a semi-structured face-to-face clinical interview described elsewhere (62). The gambling behavior variables covered included the age of onset of gambling behavior and of gambling-related problems, the average amount of money spent in a single gambling episode, the maximum amount ever bet in a single episode, and the total amount of accumulated gambling debts. In addition, the interview explored lifetime criminal activity related to GD in order to supplement the information obtained through the eighth DSM-IV-TR criterion (6). Crime-centered typologies were used to group subjects into three categories: those who conducted petty theft (the most frequent criminal behavior in our clinical population); those who committed other offenses (including counterfeiting or crimes against the public, among others); and those with multiple types of offenses.

### Statistical Analyses

Statistical analyses were carried out with Stata 13.1. Comparison between groups was based on chi-square tests (χ^2^) for categorical variables, *t*-test procedures for two mean comparisons in independent groups, and analysis of variance for mean comparisons in three or more independent groups.

The predictive capacity of impulsivity (UPPS-P raw scores) for the presence of illegal acts was based on binary logistic regression (adjusted for the covariates age of onset, GD duration, cumulate debts from gambling and GD severity). Goodness of fit was assessed through Hosmer–Lemeshow test (*p* > 0.05 was considered adequate fitting), global predictive capacity through Nagelkerke’s pseudo-*R*^2^ coefficient and global discriminative capacity through the area under the ROC curve.

Increases in Type-I error due to multiple statistical comparisons was controlled through Finner’s correction, a procedure included in Familywise error rate stepwise procedures which offers more powerful results than Bonferroni correction (63). Effect size for comparisons between groups was estimated through Cohen’s-*d* coefficient (moderate effect size was considered for |*d*| > 0.50 and good for |*d*| > 0.80), and through the 95% confidence interval (95% CI) for the logistic regression.

Since this study was planned posterior to the data recruitment, the calculation of the required sample was not possible. However, a power calculation for statistical analysis based on two independent mean comparisons was carried out with the following parameters: total sample size equal to *n* = 382, bilateral contrasts and expected mean values for the groups equal to 50 and 55 (these means were selected based on T-standardized scores commonly employed in clinical research, whose distributions include the parameters: mean μ = 50 and SD σ = 10 in community samples). Estimated power resulted in 0.983 (risk β = 0.017, less than 2%). For the chi-square test which compares two independent proportions (set at 60 and 75%), the power estimated resulted in 0.870 (risk β = 0.130).

## Results

### Sample Description

The first section of Table 1 includes the sociodemographic characteristics of the sample stratified by the presence/absence of a history of illegal behavior. Most participants were born in Spain (95.3%), had finished primary school (57.6%), were single or separated/divorced (59.2%), were employed (55.5%) and were in a middle-low to low socioeconomic status level (51.3%) (Hollingshead, Unpublished manuscript)^1^. No statistically significant differences in sociodemographic characteristics between patient groups were found.

**Table 1:** Sample description.

	No illegal acts (*n* = 279)		Illegal acts (*n* =103)										

	*n*	*%*	*n*	*%*	χ^2^	*df*	*p*						
Nationality													
Spain	267	95.7	97	94.2	0.39	1	0.533						
Other	12	4.3	6	5.8									
Education level													
Primary	163	58.4	57	55.3	1.97	2	0.373						
Secondary	96	34.4	34	33.0									
University	20	7.2	12	11.7									
Civil status													
Single or divorced	168	60.2	58	56.3	0.48	1	0.491						
With a partner (married)	111	39.8	45	43.7									
Employment													
Unemployed	119	42.7	51	49.5	1.43	1	0.231						
Employed	160	57.3	52	50.5									
Socioeconomic status													
High	5	1.8	2	1.9	2.10	2	0.349						
Mean	137	49.1	42	40.8									
Low	137	49.1	59	57.3									

**Numeric variables**	**No illegal acts (*n* = 279)**					**Illegal acts (*n* = 103)**					***T*_(df = 380)_**	**SE**	***p***
		**Min**	**Max**	**Median**	**Mean**	**SD**	**Min**	**Max**	**Median**	**Mean**	**SD**			

Age (years old)	18	75	42	42.70	14.08	19	75	39	39.73	12.25	1.89	1.47	0.059
Age of GD onset (years old)	12	70	28	30.23	12.12	13	67	26	26.79	10.07	2.49	1.27	0.013^a^
GD duration (years)	1	27	3	5.86	6.38	1	25	6	8.27	7.42	3.03	0.85	0.003^a^
Monthly income (€)	0	30,000	1,200	1,409	2,270	0	21,000	1,100	1,296	2,170	0.42	265.46	0.677
Maximum spent in a episode (€)	20	60,000	400	1,197	4,287	10	60,000	750	2,659	7,945	2.30	635.56	0.022^a^
Average spent per episode (€)	10	5,000	25	155	512	3	5,000	30	171	516	0.27	59.12	0.787
Cumulate debts (€)	0	60,000	675	6,256	12,404	0	60,000	2,175	13,348	19,148	3.95	1794.46	<0.001^a^
DSM-5 total criteria (α = 0.834)	4	9	7	6.45	2.26	4	9	8	7.77	1.33	5.58	0.24	<0.001^a^
SOGS total score (α = 0.800)	2	17	10	10.17	3.02	4	19	13	12.57	2.76	7.01	0.34	<0.001^a^

*^a^Significant comparison (0.05 level)*.

*Min, minimum; Max, maximum; df, degrees of freedom; α, Cronbach’s alpha in the sample*.

The second section of Table 1 includes GD-related variables. No differences in chronological age, monthly income, and mean amount spent per gambling episode between groups were found. However, patients who reported engaging in illegal activities endorsed a younger age of gambling onset and longer duration of GD. Patients with a criminal record also had higher GD severity levels on the SOGS as well as greater gambling-related debts.

### Comparison between Patients with and without a History of Criminal Behavior

Table 2 includes a comparison of impulsivity/personality traits, psychopathology, and substance use behaviors in patients who reported a history of engaging in illegal activity. Patients with a criminal history reported higher levels in positive and negative urgency, lack of premeditation and lack of perseverance compared to GD patients with no criminal record. GD patients who reported having committed gambling-related crimes also had higher levels of psychopathology (according to the SCL-90-R). In terms of personality traits, GD patients with a criminal record presented higher levels of novelty seeking and lower levels of self-directedness and cooperativeness compared to GD patients without a criminal record. No differences between groups were found with regards to substance use/abuse.

**Table 2:** Clinical comparison between patients with and without illegal acts.

	α	No illegal acts (*n* =279)		Illegal acts (*n* =103)		*T_(df_*_=380)_	SE	*p*	*|d|*	Power

		Mean	SD	Mean	SD					
**Impulsivity: UPPS-P subscales**										
Lack of premeditation	0.852	23.07	6.39	25.77	6.54	3.64	0.742	<0.001^a^	0.42	0.953
Lack of perseverance	0.852	21.47	5.27	23.16	6.14	2.65	0.636	0.008^a^	0.29	0.753
Sensation seeking	0.778	27.29	8.59	28.56	8.91	1.28	1.000	0.203	0.15	0.753
Positive urgency	0.851	31.01	10.44	34.46	10.14	2.88	1.195	0.004^a^	0.33	0.820
Negative urgency	0.922	32.30	7.05	34.09	7.01	2.21	0.812	0.028^a^	0.25	0.795
Psychopathology: SCL-90R										
GSI score	0.860	0.92	0.63	1.28	0.76	4.73	0.077	<0.001^a^	0.52^b^	0.997
**Personality traits: TCI-R scales**										
Novelty seeking	0.705	108.05	12.97	114.29	11.36	3.92	1.594	<0.001^a^	0.51^b^	0.974
Harm avoidance	0.808	99.12	16.76	100.50	15.85	0.72	1.906	0.470	0.08	0.888
Reward dependence	0.788	98.90	14.83	98.25	15.27	0.37	1.726	0.710	0.04	0.934
Persistence	0.885	107.94	20.46	107.46	22.51	0.20	2.427	0.844	0.02	0.946
Self-directedness	0.862	133.86	20.34	120.05	21.24	5.81	2.376	<0.001^a^	0.66^b^	0.908
Cooperativeness	0.797	132.03	14.48	126.85	18.09	2.89	1.793	0.004^a^	0.32	0.821
Self-Transcendence	0.818	60.61	13.70	63.26	15.40	1.62	1.636	0.106	0.18	0.634
**Substances: use-abuse**		***n***	**%**	***N***	**%**	**χ^2^**	***df***	***p***	***|d|***	**Power**
Tobacco use		159	57.0	59	57.3	0.00	1	0.959	0.01	0.053
Alcohol: AUDIT total		5.26	6.44	5.86	6.81	0.59	1	0.557	0.09	0.060
Other drugs: DUDIT total		3.31	7.15	3.64	6.74	0.296	1	0.768	0.05	0.060

*p includes Bonferroni–Finner correction for multiple comparisons*.

*^a^Significant comparison*.

*^b^Effect size in the moderate (|*d*| > 0.50) to high (|*d*| > 0.80) range*.

*df, degrees of freedom; |d|, Cohens’-d measuring effect size; α, Cronbach’s alpha in the sample*.

### Predictive Capacity of Impulsivity Levels on Criminal Behavior

The upper part of Table 3 includes the logistic regression measuring the predictive capacity of impulsivity levels (measured through the UPPS-P scales) on the presence of illegal acts in the entire sample. The model was carried out in two blocks/steps: the first block included and set the covariates age of onset and GD duration and second block added the five UPPS-P subscales. After adjusting for the covariates, the odds of having a history of criminal behavior was increased for patients with higher scores in the lack of premeditation and positive urgency impulsivity subscales. Goodness of fit was obtained (Hosmer–Lemeshow: *p* = 0.167), and the model showed moderate predictive capacity (the increase/change in the *R*^2^ coefficient comparing first and second block was Δ*R*^2^ = 0.12) and moderate discriminative capacity (AUC = 0.68).

**Table 3:** Predictive capacity of impulsivity profile (UPPS-P scores) on the presence of illegal acts: logistic regression adjusted for age of gambling disorder onset and GD duration.

	*B*	SE	Wald	*p*	OR	95%CI (OR)	
**Covariates**							
Age of GD onset	−0.018	0.012	2.173	0.140	0.982	0.959	1.006
GD duration (years)	0.052	0.018	8.490	0.004^a^	1.053	1.017	1.090
**UPPS-P**							
Lack of premeditation	0.059	0.026	5.261	0.022^a^	1.061	1.009	1.116
Lack of perseverance	−0.018	0.029	0.375	0.540	0.982	0.928	1.040
Sensation seeking	0.003	0.016	0.036	0.850	1.003	0.973	1.034
Positive urgency	0.044	0.018	5.639	0.018^a^	1.045	1.008	1.083
Negative urgency	−0.017	0.028	0.363	0.547	0.983	0.931	1.039
Fitting indexes: H-L; Δ*R^2^*; AUC	0.167	0.121	0.684				

*^a^Significant parameter. N = 382*.

*H-L, Hosmer–Lemeshow test (*p*-value); Δ*R*^2^, increase in the Nagelkerke’s *R*^2^ coefficient comparing blocks 1 and 2; AUC, area under the ROC curve*.

Table S1 in Supplementary Material contains a new predictive model including also two additional GD-related measures as covariates into the first block: cumulate debts and disorder severity (SOGS total score). In the resulting logistic predictive regression, UPPS-P positive urgency raw score remained a significant predictor.

### Comparison Based on Type of Illegal Act

Table 4 contains a comparison between the *n* = 103 GD patients who reported a history of illegal activity based on the type of crime(s) committed (theft, other, or multiple). A number of patients (*n* = 25) chose not to specify which type of gambling-related illegal act they committed and these patients were excluded from this analysis. Patients who reported committing multiple types of illegal acts obtained the highest means in cumulate debts due to gambling, and higher GD severity levels according to the SOGS.

**Table 4:** Clinical comparison for patients based on type of illegal act committed.

	Petty Theft		Other		Multiple		Petty theft vs. Other		Petty theft vs. multiple		Other vs. multiple	
						
	*n* =38		*n* =29		*n* =11	
						
	Mean	SD	Mean	SD	Mean	SD	*p*	*|d|*	*p*	*|d|*	*p*	*|d|*
**Gambling: duration-severity**												
Age (years-old)	38.53	15.90	40.45	8.57	38.45	6.12	0.536	0.15	0.987	0.01	0.655	0.27
GD onset (years-old)	26.86	12.82	26.05	7.14	24.22	8.38	0.760	0.08	0.502	0.24	0.650	0.23
GD duration (years)	7.92	7.61	8.25	6.95	11.00	9.15	0.862	0.05	0.277	0.37	0.346	0.34
Maximum spent/episode (€)	1,493	2,645	3,657	1,1055	1,503	2,911	0.220	0.27	0.997	0.00	0.393	0.27
Mean amount spent/episode (€)	110	183	326	921	79	113	0.134	0.33	0.877	0.20	0.233	0.38
Cumulate debts (€)	3,083	8,314	21,593	23,680	26,380	26,480	0.001^a^	1.04^b^	0.001^a^	1.19^b^	0.491	0.19
DSM-5 total criteria	7.68	1.36	7.55	1.48	7.36	1.36	0.703	0.09	0.507	0.24	0.706	0.13
SOGS total score	11.84	2.63	12.45	3.42	14.27	1.42	0.389	0.20	0.015^a^	1.15^b^	0.074	0.70^b^
**Substances: use-abuse**	***n***	***%***	***n***	***%***	***N***	***%***	***p***	***|d|***	***p***	***|d|***	***p***	***|d|***
Tobacco use	20	52.6%	16	55.2%	5	45.5%	0.836	0.05	0.675	0.14	0.583	0.20
Alcohol use-abuse	6	15.8%	5	17.2%	2	18.2%	0.874	0.04	0.850	0.06	0.944	0.02
Other drugs use-abuse	9	23.7%	5	17.2%	1	9.1%	0.520	0.16	0.290	0.40	0.519	0.24

*|d|, Cohens’-d measuring effect size*.

*p includes Bonferroni–Finner correction for multiple comparisons*.

*^a^Significant comparison*.

*^b^Effect size in the moderate (|*d*| > 0.50) to high (|*d*| > 0.80) range*.

## Discussion

This study analyzed differences in impulsivity and personality traits between treatment-seeking GD patients who committed illegal acts and those who did not. Moreover, we sought to examine the interplay between criminal typology, sociodemographic features, and psychological variables.

Regarding the multidimensional nature of risk factors for engaging in crime, as suggested by previous studies, sociodemographic (especially gender and age) (64), education (65), and economic factors (such as socioeconomic status) (12) were determinants of the incidence of crime. In Western populations, the association between age and crime mainly follows a bell-shaped pattern, known as the age-of-crime curve, showing a reduction in criminal activity as an individual progress into adulthood (66, 67). Surprisingly, no differences were found between GD patients who committed crimes and those who did not, even though we hypothesized that the GD patients with a criminal record would be younger. It is worth noting, however, that our sample was made up patients voluntarily seeking treatment for GD and that we did not explore at what age these patients began engaging in illegal activity to finance their gambling behavior.

On the other hand, earlier studies have shown that education may counteract the risk of committing crimes, being that those with a higher level of education have higher expectations regarding the amount of income they can derived from legal ventures (65). Moreover, the inverse relationship between social stratification and delinquency turns out to be one of the main points of interest in criminology (68). However, contrary to expectations, this study did not find significant differences between groups in years of schooling. These results may partly be explained by the fact that our sample consisted of gamblers who sought treatment of their own volition and therefore our results are not necessarily representative of gamblers as a whole. Similar issues arise in the case of substance abuse as most individuals report first using drugs at a younger age and not seeking treatment until they are often much older (69). In this vein, an additional explanation could be that only crimes related to gambling behavior have been evaluated and those subjects whose main clinical problem was exclusively GD were included in the study.

Keeping with our hypothesis, patients who committed GD-related crimes reported greater GD severity, higher maximum bets and more cumulated debts in comparison with those who did not. This result dovetails with previous studies also reporting that GD patients with gambling-related crimes experienced more severe gambling symptoms than did other gamblers (15, 16, 21, 27). These findings suggest that greater gambling-related economic expenditures (more money spent during gambling episodes and more overall gambling-related debts) would increase an individual’s likelihood of resorting to illegal behaviors in order to obtain money rapidly and, consequently, to be able to continue addictive-like gambling behavior.

Another finding to emerge from the present study is the difference in age of onset of GD between both groups, showing earlier onset in the illegal acts group. In our study, the measure to determine “onset” referred to the moment when the patients identified that gambling behavior had become harmful and uncontrollable. In this vein, previous studies showed that several factors are associated with early GD onset, including higher trait impulsivity and substance use disorders (70, 71).

Relatedly, our stepwise analyses identified both positive urgency and lack of premeditation to be predictors of the presence of illegal activity in GD patients. Both of these impulsivity traits have been found to commonly be higher in younger individuals and could potentially be seen as a risk factor, though longitudinal are needed to support this claim (35, 72). With regards to personality traits, GD patients with a history of criminal behaviors also reported lower levels of self-directedness. Self-directedness is characterized by possessing an external locus of control and, therefore, encountering more difficulties in planning, decision-making and achieving goals (56). This finding is consistent with other studies highlighting low levels of self-directedness across psychiatric disorders (73–75). Contrary to our hypothesis, no differences were found in substance use/abuse prevalence between GD patients who did and did not report committing gambling-related crimes. This may be partly due to the fact that we only assessed current substance-use patterns in our sample and that all of our patients were voluntarily seeking treatment.

Although some demographic risk factors have been identified for criminal recidivism (in particular gender, age, and race), in recent years there has been much debate about whether sociodemographic factors in themselves can fully account for the complexity behind reoccurring criminal behaviors (76, 77). In our sample, GD patients who had committed multiple offenses endorsed greater GD severity levels and greater amounts of gambling-related debts. These results coincide with other studies supporting the existence of subgroups of gamblers that are distinguishable according to their gambling-related criminal behaviors (27).

### Ethical Issues Raised by the Study

Our analysis seems to prompt at least two important moral issues. The first pertains to autonomy. If GD patients with a history of criminal behavior tend to report lower levels of self-directedness, it can be argued that their capacity for autonomous action is, in some sense, diminished. This is important because autonomy is tied to responsibility. The less autonomous an individual is, the less responsible we hold them for their actions. If GD patients who engaged in illegal acts tend to display lower levels of autonomy, we should take this fact into account when making attributions of responsibility. This overlaps with our previous discussion of the Spanish court system and its *de facto* concern for gambling-related instances of reduced free will. The second issue arises once we realize that both positive urgency and lack of premeditation are predictors of the presence of illegal activity in GD patients. Given the serious risk of adding stigmatization to this population, we should set a high bar in terms of predictive value before using such variables as proxy for policy-making. And if this becomes unavoidable, then efforts should be made to minimize the risk of stigmatization as much as possible. However, given the self-acknowledged limitations of this analysis, this should be considered (i.e., whether such predictors are robust enough for determining future policies) an open question.

### Limitations

Our results must be interpreted in light of their limitations. The main weakness of this study was that exploring criminal behaviors through self-report in a clinical interview and not administering a validated psychometric instrument may have generated false negatives and limited the thoroughness of the obtained information. Second, our sample was made up exclusively of male GD patients, and taking into account that male gender is one of the indicators most associated with gambling-related crimes (12), the generalizability of the results to other populations is discouraged (78). Finally, the present study was focused exclusively on criminal behaviors carried out with the aim of financing debts derived from gambling or ensuring the continuity of gambling behavior. Future studies should consider the full scope of illegal behaviors carried out by GD patients, even those not directly related to gambling.

## Conclusion

This study provides greater empirical understanding of the associations between GD, impulsivity, and criminal behavior. Our findings suggest that high levels of trait impulsivity, especially lack of premeditation and positive urgency, are predictors of the occurrence of crime in those who gamble. Further research should be undertaken to examine the effectiveness of interventions targeting impulse traits and recidivism risk management in gambling populations. Such detailed information would be useful in improving GD treatment and harm reduction interventions.

## Author Contributions

GM-B, TS, FF-A, RG, JM, and SJ-M designed the experiment based on previous results and the clinical experience of M-TN, AC, MB, LM, AP-G, NA, MG-P, CV-A, and NM-B. RG, GM-B, TS, FF-A, and SJ-M conducted the experiment, analyzed the data, and wrote a first draft of the manuscript. SJ-M, TS, GM-B, RG, PM, and FF-A further modified the manuscript.

## Conflict of Interest Statement

The authors declare that the research was conducted in the absence of any commercial or financial relationships that could be construed as a potential conflict of interest.

## Footnotes

^1^Hollingshead, A. A. *Four-factor index of social status*. Unpublished manuscript, Yale University, New Haven, CT (1975).
