# Supplementary material for: Gambling and Impulsivity Traits: A Recipe for Criminal Behavior?
Source: Front Psychiatry. 2018 Jan 29;9:6. doi: 10.3389/fpsyt.2018.00006 (PMC5796889; doi:10.3389/fpsyt.2018.00006)
Supplement: Supplementary file 1 [file Table_1.docx]

Table S1. Predictive capacity of impulsivity profile (UPPS-P scores) on the presence of illegal acts: logistic regression adjusted for the age of GD onset, cumulate debts, GD severity and GD duration.

| Covariates: Age of GD onset | -0.020 | 0.015 | 1.970 | .160 | 0.980 | 0.952 | 1.008 |
| --- | --- | --- | --- | --- | --- | --- | --- |
| GD duration (years) | 0.048 | 0.021 | 5.557 | .018 | 1.050 | 1.008 | 1.093 |
| Cumulate debts (€) | 0.000 | 0.000 | 7.737 | **.005** | 1.000 | 1.000 | 1.000 |
| GD severity: SOGS-total | 0.236 | 0.054 | 18.788 | **<.001*** | 1.266 | 1.138 | 1.408 |
| UPPS-P: Lack of premeditation | 0.043 | 0.029 | 2.218 | .136 | 1.044 | 0.987 | 1.104 |
| Lack of perseverance | -0.006 | 0.033 | 0.031 | .860 | 0.994 | 0.933 | 1.060 |
| Sensation seeking | 0.006 | 0.018 | 0.105 | .745 | 1.006 | 0.972 | 1.041 |
| Positive urgency | 0.044 | 0.021 | 4.532 | **.033*** | 1.046 | 1.004 | 1.089 |
| Negative urgency | -0.009 | 0.032 | 0.087 | .768 | 0.991 | 0.930 | 1.055 |
| *Fitting indexes: H-L; ΔR^2^; AUC* | *.265* | *.050* | *.777* |  |  |  |  |

*Note.* H-L: Hosmer-Lemeshow test (*p-value).* ΔR^2^: increase in the Nagelkerke’s R^2^ coefficient comparing blocks 1 and 2. AUC: area under the ROC curve.

***Bold: significant parameter. *N=382.*
